# Causes and Consequences of Age-Related Changes in DNA Methylation: A Role for ROS?

**DOI:** 10.3390/biology3020403

**Published:** 2014-06-18

**Authors:** Franka J. Rang, Johannes Boonstra

**Affiliations:** 1 Science Department, University College Utrecht, Campusplein 1, 3584 ED Utrecht, The Netherlands; E-Mail: franka.j.rang@gmail.com; 2 Faculty of Science, University of Utrecht, Padualaan 8, 3584 CH Utrecht, The Netherlands

**Keywords:** DNA methylation, methylcytosine, aging, reactive oxygen species, mechanisms

## Abstract

Recent genome-wide analysis of C-phosphate-G (CpG) sites has shown that the DNA methylome changes with increasing age, giving rise to genome-wide hypomethylation with site‑specific incidences of hypermethylation. This notion has received a lot of attention, as it potentially explains why aged organisms generally have a higher risk of age-related diseases. However, very little is known about the mechanisms that could cause the occurrence of these changes. Moreover, there does not appear to be a clear link between popular theories of aging and alterations in the methylome. Some of the most fruitful of these theories attribute an important role to reactive oxygen species, which seem to be responsible for an increase in oxidative damage to macromolecules, such as DNA, during the lifetime of an organism. In this review, the connection between changes in DNA methylation and these reactive oxygen species is discussed, as well as the effect of these changes on health. Deeper insights into the nature, causes and consequences of the aging methylome might provide a deeper understanding of the molecular mechanisms of aging and eventually contribute to the development of new diagnostic and therapeutic tools.

## 1. Introduction

For thousands of years, mankind has been in search of the fountain of youth. However, over the past century, the focus of this search has shifted and researchers are now fervently investigating the source of aging [1,2,3]. Recently, more attention has been given to the role of DNA methylation. In particular, there appear to be extensive shifts in the pattern of DNA methylation that occur over the lifetime of an organism [4,5,6]. Since DNA methylation plays a central role in gene regulation and expression, such changes might influence the behavior of the cell and potentially contribute to the aging process. 

One way in which altered DNA methylation may play a role is by mediating the increased risk for certain pathologies that are typical for aged organisms. The most striking alteration in the methylome appears to be the emergence of regions of age-associated hyper- and hypomethylation. Interestingly, these age-related shifts carry a considerable resemblance to the methylation profile of diseases typical of old age, such as cancer [5,7]. Based on these observations, it is now believed that the age-related shifts in patterns of DNA methylation are a main contributor to the process of carcinogenesis [8,9]. Although it is still unclear whether these changes also result in a shortening of the organism’s lifespan, this does provide a mechanism through which the risk of certain pathologies may increase with age. 

Due to such research into the extent and consequences of alterations in the methylome, the link between aging and DNA methylation has been increasingly recognized, and it is now widely established that changes to DNA methylation that occur with age contribute to age-related diseases. However, although the occurrence and consequences of DNA methylation modifications have been and are being extensively researched, its causes remain shrouded in mystery. Despite some correlations between genetic polymorphisms and differential aging of the methylome [10,11], it currently remains unclear how differential patterns of DNA methylation observed with increasing age relate to existing theories of aging. 

One of the most widely studied theories of aging is the mitochondrial theory of aging, which was first proposed by Harman in 1956 [12] and states that free radicals that arise within the cell are the main cause of cellular damage and subsequent aging. In the current version of this theory, increasing levels of reactive oxygen species (ROS) are thought to be responsible for the observed oxidative damage to cellular components that arise with age [13]. Since both levels of ROS and DNA methylation seem to change with age, it is of interest to study a potential link between them. Indeed, ROS have been shown to be able to affect DNA methylation in numerous studies [14,15,16]. As such, alterations in the methylome may provide one avenue through which ROS exert their deleterious effects. 

The goal of this review is to explore the link between age-related changes in DNA methylation and the increase of oxidative stress associated with age. To this purpose, an overview will be given of the observed differences in DNA methylation as found by multiple statistical studies that compare the methylation status of a vast number of C-phosphate-G (CpG) sites from a large cohort of subjects. Subsequently, several proposed mechanisms of ROS-induced DNA methylation based on experimental results and chemical insights will be discussed. Such insights are of importance to understand the processes of aging and the incidence of age-related diseases and can provide a link between the theory and phenomenon of aging. Finally, the effect of these changes in the methylome on health span will be discussed. 

## 2. Basics of DNA Methylation

DNA methylation refers to the addition of a methyl (-CH_3_) group to the 5-carbon atom of a base in DNA, most commonly a cytosine residue linearly linked to a guanine residue through a phosphate group. Due to the symmetry of these CpG sites, methylated cytosines come in pairs, with one on each strand. This provides a blueprint through which methylation can be conserved during DNA replication, where each parental CpG site induces methylation of the corresponding nascent strand. CpG sites are relatively rare in the genome, except for certain genomic locations where CpGs occur in high concentration, so called CpG islands (CGIs) [17]. In mammals, CGIs remain generally unmethylated, whereas the majority of all other CpG sites are methylated [17]. Such CGIs mostly occur in promoter regions, although they can also be found in intragenic regions. In those cases where promoter CGIs are methylated, the corresponding gene is usually stably repressed. This has led to the general view that an increase in DNA methylation indicates higher levels of gene repression [18]; although this notion has been challenged by the observation that intragenic CpG methylation is positively correlated with active gene expression [19], as reviewed by Suzuki and Bird [20]. 

As becomes evident from the above, the function of DNA methylation depends largely on its context. Methylation of CpG sites at promoters and intragenic regions seem to be important in gene repression and activation, respectively, suggesting that methylation inhibits initiation of transcription but not elongation [21,22]. This is in line with the supposed role of DNA methylation of silencing repetitive elements within genes [23], such as retroviruses and LINE1 elements. In this way, the methylation blocks transcription of these elements, while allowing elongation along the gene. Other proposed functions of DNA methylation include the regulation of splicing [24], modulation of the activity of enhancers [25,26] and maintaining chromosomal stability [27,28].

**Figure 1:**
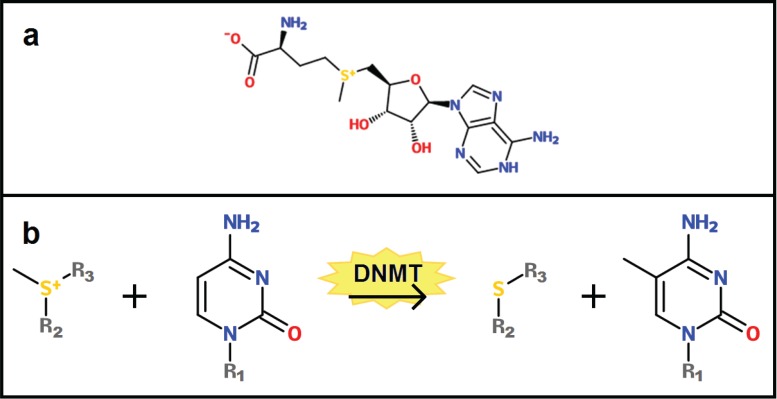
(**a**) The chemical structure of S-adenosyl methionine (SAM), which functions as a methyl donor during the methylation of a cysteine residue. (**b**) During DNA methylation, a DNA methyltransferase (DNMT1, DNMT3a or DNMT3b) catalyzes the transfer of a methyl residue from the sulfur atom of SAM to the 5-carbon atom of a cytosine base. In the figure, R1 represents the rest of the DNA molecule to which the cytosine is attached, and R2 and R3 represent the rest of SAM.

In order for these functions to be properly fulfilled, the DNA methylome needs to be accurately established during development and maintained after birth. The most important players in these processes are the DNA methyltransferases (DNMTs), which catalyze the addition of the methyl group to cytosine [29,30]. In humans, there are four different DNMTs: DNMT3a and DNMT3b are the *de novo* methyltransferases that are responsible for establishing the DNA methylome during development [31], although it has recently been found that these enzymes also play a role in methylation maintenance [32]; DNMT1 is the main enzyme responsible for the maintenance of the methylation pattern during DNA replication [33,34]; and DNMT3L, lacking a catalytic domain, stimulates the activity of DNMT3a and DNMT3b by binding these enzymes, thereby increasing their interaction with DNA and the methyl donor [35]. This methyl donor is a small molecule called S‑adenosyl methionine (SAM) (see Figure 1a), which is made from adenosine triphosphate (ATP) and methionine [36]. The methyl group on SAM is very reactive and can be transferred to the fifth carbon atom of cytosine by the catalytic action of the DNMTs (see Figure 1b). 

## 3. Changes in the DNA Methylome with Age

Since all cells contain the same DNA (except for T and B cells), epigenetic control is of paramount importance in establishing the vast spectrum of different cell types present in an organism. It has been shown that DNA methylation may contribute to this diversity through differential methylation patterns across tissues [37]. Soon after the first evidence for tissue-specific DNA methylomes and a role for DNA methylation in differentiation [38,39], it was shown that tumors also display a unique methylation pattern with high levels of hypomethylation [40]. Since age is a major demographic risk factor for many cancers, research has since been conducted on the aging methylome and its link to cancer [8,9,41,42] and other age-related diseases, such as Alzheimer’s disease [43]. In this section, the changes in DNA methylation patterns with age will be discussed, whereas the link to disease will be discussed in a later section. 

Over the past decade, many studies have been performed that compared the methylomes of populations varying in age [4,6,44], the largest of which comprised the analysis of more than 450,000 CpG markers of whole blood in a population of 656 people [44]. Although the results depended on the genomic location and number of the CpG loci studied, these studies showed that a considerable percentage (1.5%–29%) of CpG sites is affected by age. From these data, a general bimodal pattern of age-dependent differential DNA methylation can be observed, in which a genome-wide loss of methylation accompanies site-specific incidences of hypermethylation [4]. Several studies have shown that these hypermethylation events correspond to CpGs that are located within CpG islands, whereas the hypomethylation mostly occurs outside the CGIs [4,5,6]. It’s interesting to note that even though DNA methylation patterns generally differ between tissues, this trend appears to be the same across different cell types [6]. This observation might be related to the fact that most CpG sites are methylated, with the exception of loci within CGIs, which usually remain unmethylated. Due to this strict division, any change in methylation would most likely result in hypermethylation of CpG or hypomethylation of other genomic regions.

Importantly, there appears to be a link between genotype and changes in DNA methylation. Christensen *et al.* [4] showed that 5% of the CpG loci affected by age was associated with a single nucleotide polymorphism (SNP). Such genetic variants that influence the methylation status of age‑associated CpGs are called methylation quantitative-trait loci (meQTLs) [4]. Some of these meQTLs were located in genes previously associated with longevity and aging. The researchers therefore concluded that in a small portion of genes, genetic and methylation effects may impart age-related phenotypes, either through independent mechanisms or through genotype-phenotype associations mediated by DNA methylation. 

Even more striking evidence for a link between genotype and age-associated methylation comes from research conducted by Hannum *et al.* [44]. In their study, these researchers used 71 strongly age-dependent methylation markers (CpGs) to set up a model to determine a person’s methylomic age. By combining the methylomic ages and chronological ages of their population, they subsequently determined the ‘apparent methylomic aging rate’ of individuals. Interestingly, there appeared to be a few meQTLs that were not only associated with age, but also with this aging rate. One of these meQTLs concerned a missense mutation in GTPBP10 and was associated with a CpG methylation marker in the gene STEAP2. This gene is known to play a role in maintaining homeostasis of iron and copper, which are important components of the mitochondrial respiratory chain. It has been shown that perturbations in iron concentrations can cause oxidative stress in mammalian cells [44,45,46]. Hannum *et al.* [44] also found that changes in the methylome with age were indicative of functional changes in gene expression. Together, this research offers a potential link between oxidative stress and age-related DNA methylation. 

In addition to genetic elements, environmental factors have been found to influence changes in DNA methylation with age. In light of this review, it is most interesting to note that factors such as tobacco smoke have been shown to influence the changes of the methylome over time. Numerous studies have demonstrated that cigarette smoke is significantly associated with hypermethylation of the promoter regions of cancer associated genes [47,48,49,50,51], as well as with shifts in general methylation profiles [52,53,54]. Similarly, asbestos has been found to induce hypermethylation of a range of CpG loci [4,55,56]. Moreover, these factors are also known to induce oxidative stress [57,58], and thus provide support for the connection between free radical production and differential DNA methylation. 

## 4. Influence of ROS on DNA Methylation

Although observations such as described in the previous sections hint at a link between aging, an increase in ROS, and changes in DNA methylation, the mechanisms that might be responsible have remained elusive. In the following section, ROS, their influence on DNA methylation, and the potential mechanisms mediating this influence will be discussed.

### 4.1. Reactive Oxygen Species in Aging

Over the years, numerous theories of aging have been proposed that attribute important roles to somatic mutations [1], dysfunctional transcription resulting in aberrant proteins [2], and telomere shortening [3]. However, the theory that has gotten the most attention was proposed by Harman in 1956 as the free radical theory of aging [12]. This theory proposes that free radicals formed by endogenous processes within the cell cause damage to cellular components and thus cause the aging process. Harman later refined his theory to the mitochondrial free radical theory of aging (MFRTA), which postulates that the mitochondria are the main source of such damaging agents, *i.e.*, reactive oxygen species [59]. Since then, this theory was further developed to include all forms of ROS and is now also referred to as the oxidative stress theory of aging [13]. The central idea of all of these theories is that there is a chronic imbalance between oxidizing agents and antioxidants, which increases with age. This imbalance, referred to as oxidative stress, results in the damaging of macromolecules and a decline in the function of cellular processes. In turn, this deterioration can further unbalance the system and increase oxidative stress, resulting in a vicious circle of accumulating damage.

Recently, the MFRTA has been disputed as some experimental findings are not in line with the predictions made by this theory, as reviewed and argued by Lapointe and Hekimi [60]. For example, several studies showed that the overexpression of antioxidants in model organisms did not increase their life span [61,62]. The causative role of increased ROS production in aging is thus being questioned. However, the observations that oxidative stress, ROS overproduction and oxidative damage increase with age remain [63,64,65]. Some have proposed that this correlation may imply that these events are a consequence rather than the primary cause of aging, and still play a role in the functional deterioration of aging [66]. Such considerations are supported by the emerging role of ROS as important players in normal cellular processes, such as signal transduction in response to growth factors [67,68], antigen-specific T-cell activation [69], maintenance of hippocampal stem and progenitor cells [70], and autophagy [71,72]. Yet, it remains unclear what else would be the primary cause of aging. 

In concord with this theory, Salmon *et al.* [73] proposed that ROS may not exert a direct influence on lifespan (*i.e.*, the maximum age an organism can attain), but rather on health span (*i.e.*, the maximum disease-free age that an organism reaches). This hypothesis is based on the observation that decreased expression of antioxidants only rarely led to a decrease in lifespan, but resulted quite often in the early onset of age-related diseases (extensively reviewed by Salmon *et al.* [73]). It is thus possible that oxidative stress affects the health of an organism without necessarily altering its lifespan. 

Although these theories vary substantially in the role they ascribe to ROS, they all postulate that ROS levels increase with age and eventually lead to damage within the cell. When investigating the role of ROS in the aging process and its influence on both health and lifespan, it is thus important to not only consider the phenomenon itself, but also to look at its consequences. ROS are very reactive and as such can oxidize many components within the cell, including mitochondrial DNA (mtDNA), nuclear DNA (nuDNA), lipids, and proteins [74]. Such reactions alter the structure of the macromolecules and eventually give rise to the increase in oxidative damage during aging. In other words, although ROS might not play the causative role that has been ascribed to them for some decades, it is still likely that they mediate many of the effects that are associated with aging.

### 4.2. Formation, Regulation and Function of ROS

ROS are potentially highly reactive molecules that contain an oxygen atom, such as the superoxide radical (·O_2_-) and hydrogen peroxide (H_2_O_2_). These reactive moieties can arise from both endogenous and exogenous sources. The endogenous sources include cytochrome P450-dependent oxygenases, which are responsible for the oxidation of numerous organic compounds in the body [75]; metabolic enzymes in peroxisomes [76,77]; and NADPH oxidases that are present in phagocytic cells and can produce a respiratory burst of superoxide in response to pathogens [78,79]. Although there are many other sources of ROS, an important one seems to be oxidative phosphorylation within mitochondria. This important observation led Harman to formulate his mitochondrial free radical theory of aging [59]. During oxidative phosphorylation, an electron is passed through the electron transport chain in a series of redox reactions. The final electron acceptor is dioxygen, which is normally reduced to water. However, in 1%–2% of the cases, dioxygen is partially reduced to form ROS [80,81]. Further reduction reactions can subsequently give rise to hydrogen peroxide. In addition, there are several environmental factors that can induce the production of ROS, including pollutants and tobacco smoke [82], diets including plant food rich in phenolic compounds [83], iron salts [84], UV [85] and ionizing radiation [86].

Under normal physiological conditions, the production of ROS can be controlled and neutralized by a variety of antioxidant enzymes, such as superoxide dismutase [87], as well as several non-enzymatic molecules, such as glutathione and vitamins A, C and E [88]. Moreover, it has been shown that ROS also play an important role in normal cell signaling, such as in protein phosphorylation [89], signal transduction in response to growth factors [67,68], and more recently in the activation of antigen‑specific T-cell activation [69] and maintenance of hippocampal stem and progenitor cells [70]. However, if there is a lack of antioxidants or an excess of ROS, the balance is lost and the cell goes into a state of oxidative stress, which can disrupt normal cell signaling, as well as induce damage to cell organelles and the genome [90,91]. 

### 4.3. ROS-Induced Changes in DNA Methylation

Although there is still discussion about the role of ROS in the aging process, it is generally accepted that ROS levels increase with age and eventually lead to damage within the cell. This is a result of the observation that both ROS levels [92,93] and oxidative damage [64,94,95] increase with age in several tissues. The idea that ROS might influence the DNA methylome emerged from observations that cancer cells display vast shifts in methylation patterns and are often in a state of oxidative stress [96]. In agreement with this notion, ROS has been shown to be responsible for epigenetic changes in several cancer models [14,15,16,97]. Also, in other situations, an increase in ROS has been identified as a potential cause for changes in the methylome. For example, Tunc and Tremellen [98] showed that treatment with antioxidants could positively influence DNA methylation in sperm of infertile men. Since oxidative stress also increases with age, it seems probable that ROS may also affect DNA methylation in healthy cells.

Over the past twenty years, chemical, molecular and cellular studies have provided some insight into factors mediating these changes. However, a unifying mechanisms explaining both the instances of hyper- and hypomethylation has been lacking. Here, several processes that are thought to play a role are discussed.

### 4.4. Mechanisms of ROS-Induced Changes in DNA Methylation

#### 4.4.1. ROS-Induced DNA Damage Influences DNMT Activity

Due to the reactive nature of ROS, they cause the oxidation of macromolecules within the cell, most notably DNA. The oxidation of DNA results in a variety of damaged sites, including 8-hydroxyguanine (8-OHdG), which is the most commonly produced base lesion and often used as a measure of oxidative damage in DNA [74]. Such damage does not only result in an increase in the mutation frequency [74], but also exerts an influence on the DNA methylation of nearby cytosine bases. 

As early as 1994, *in vitro* experiments were conducted to show that the presence of 8-OHdG negatively affects methylation of adjacent sites [99]. Shortly after, this result was reproduced with the additional discovery that this influence was exerted on target cytosines one or two base pairs away from the damaged guanine [100]. It was suggested that oxidative damage on the nascent strand could thus lead to inhibition of DNA methylation [100]. Additionally, other oxidative damage may also contribute to the loss of DNA methylation. It has been shown that 5-hydroxymethylcytosine (5hmC) could impede faithful transmission of methylation patterns [101]. This modified base is the result of hydroxylation of methylcytosine, which can occur through attack by ROS. In their research, Valinluck and Sowers [101] showed that such hydroxylation results in >90% decrease in methylation of the target cytosine. Together, these observations provide a mechanism through which DNA methylation may decrease in subsequent rounds of cell division, slowly giving rise to a methylome characterized by general hypomethylation. 

Interestingly, over the past few years, it has become evident that the formation of 5hmC from 5mC can also be actively catalyzed by enzymes within the cell [102]. Moreover, it seems to commonly occur within some tissues, most notably bone marrow and neuronal tissue [103,104,105]. It is not said, however, that all hydroxymethylated CpG sites result in incorrect methylation patterns. The enzymatically controlled hydroxylation of 5mC appears to be a carefully regulated mechanism, largely limited to specific tissues and stages in development [105]. Oxidative damage could thus cause perturbations in this carefully maintained mechanism. In addition, incorrectly hydroxylated 5mC might be restored to unmethylated cytosine by repair mechanisms that are speculated to be responsible for active demethylation in undividing cells [106]. As of yet, little is known about the details and frequency of occurrence of these processes and research is hampered by the lack of large-scale analytical techniques that are capable of identifying genome-wide hydroxymethylation patterns. Moreover, current methods for establishing genome-wide methylome profiles are incapable of differentiating between hydroxymethylated and methylated cytosines [107]. Since 5hmC is thought to have a role distinct from 5mC and interacts differently with methyl-CpG binding proteins [108,109], it is thus hard to gauge what the influence of ROS-induced hydroxymethylation is on the epigenome and consequently the cell. 

#### 4.4.2. ROS as Catalysts of DNA Methylation

A potential mechanism by which ROS are able to induce DNA hypermethylation was recently proposed by Afanas’ev [110]. Under normal conditions, the conversion from cytosine to 5-methylcytosine is mediated by DNMTs, which catalyze the transfer of a methyl group from SAM to the nucleotide (see Figure 1b). The most important catalytic step in this reaction involves the binding of a negatively charged cysteine residue to the carbon adjacent to the atom that is to be methylated [111,112]. This addition makes the C-5 atom more negative and thus gives it nucleophilic properties. The S-atom carrying the methyl group in SAM is positively charged and is thus capably of reacting with the nucleophilic C-5 atom. However, according to Afanas’ev, the catalytic role of DNMT can also be fulfilled by superoxide.

**Figure 2:**
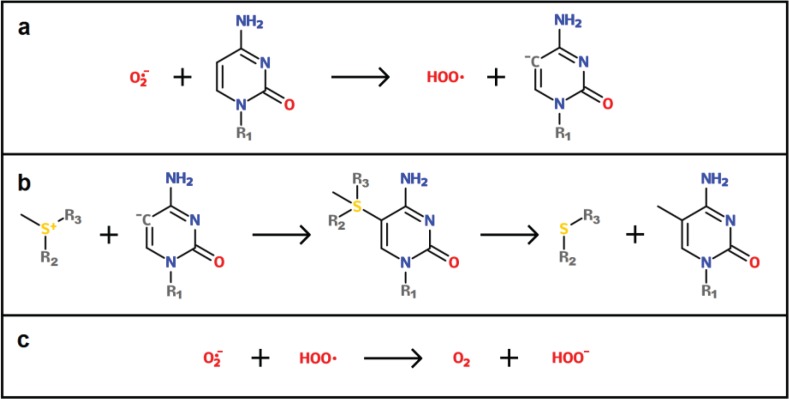
(**a**) A superoxide molecule deprotonates the 5-carbon atom of a cytosine base, leaving the carbon atom with a negative charge. R1 forms the rest of the DNA molecule to which the cytosine is attached. (**b**) The positively charged sulfur atom of SAM reacts with the C-5 atom. This reaction first yields a structure where SAM is covalently linked to cytosine, but then continues to form the two final products. R2 and R3 represent the rest of the SAM molecule. (**c**) Concurrently, the radical created during reaction (a) now reacts with another superoxide molecule, giving rise to dioxygen and hydroperoxyl. (Based on Afanasev [110]).

In this model, a superoxide molecule directly reacts with the C-5 atom and deprotonates it (see Figure 2a). After this reaction, the C-5 atom is negatively charged and can thus easily react with the positively charged S-atom of SAM, yielding the methylated cytosine (see Figure 2b). Simultaneously, the radical created during the first step can react with another superoxide molecule in order to create two non-radical molecules, *i.e.*, oxygen and hydroperoxyl (see Figure 2c).

This mechanism could potentially account for the observed cases of ROS-induced DNA hypermethylation in promoter regions. The fact that these regions are most commonly unmethylated compared with the rest of the genome could explain how this stochastic, unspecific mechanism results in site-specific hypermethylation. However, more carefully controlled experiments are necessary in order to test the capacity of ROS to induce methylation of cytosine in the presence of SAM and absence of DNMTs.

#### 4.4.3. ROS-Induced Differential Binding of a DNMT Containing Complex

A ROS-induced mechanism that may explain both the observed regions of hypomethylation and promoter-specific hypermethylation was proposed by O’Hagan *et al.* [113]. Although their research was conducted in the context of cancer cells, these findings might be extended to aging as well, since both events correlate with increased levels of ROS. Upon treatment with H_2_O_2_, they found that large complexes containing DNMT1 and DNMT3b were formed. Next to the DNMTs, these complexes contained Sirtuin1 (SIRT1) and members of the polycomb repressive complex 4 (PRC4). SIRT1 is a histone deacetylase that is recruited to sites of DNA damage and can deacetylate other proteins involved in the DNA damage [114]. In addition, SIRT1 has been shown to interact with DNMT1 [115]. An important member of the PRC4 complex is Enhancer of Zeste protein-2 (EZH2), a histone methylase that can interact with both DNMT1 and DNMT3b, facilitating the binding of these enzymes to the EZH2 target promoter. Although DNMTs have previously been shown to interact with polycomb repressive complexes in the context of gene silencing [116], this specific complex seems to respond to H_2_O_2_-induced DNA damage. Interestingly, DNMT1 has previously been shown to be recruited to sites of DNA damage, supposedly in order to restore epigenetic modifications [117]. Moreover, both DNMT1 and SIRT1 have also been shown to be recruited to double strand breaks *in vitro* [118], potentially explaining why the larger complex localizes at sites of H_2_O_2_-induced damage. 

O’Hagan *et al.* [113] found that upon treatment with H_2_O_2_, the large complex containing SIRT1, the DNMTs and members of PRC4 became enriched in areas in the genome with high levels of transcription and CpG content, especially close to transcription start sites. Conversely, a loss of these silencing complexes was observed in areas that have low transcriptional activity and CpG content. Importantly, it was shown that increased binding of this complex was indeed capable of eliciting functional changes, including histone mark changes, reduction of nascent transcription levels and increases in DNA methylation [113].

O’Hagan *et al.* [113] provided a potential explanation for the higher binding of the silencing complex to CpG islands compared to non-CpG islands. From the data, it appeared that the tight binding of members of the complex to chromatin was associated with DNA damage and/or repair. This observation was supported by the fact that 8-oxoguanine DNA glycosylase (OGG1) enhanced DNMT1 binding to the chromatin. OGG1 is an enzyme responsible for the excision of 8-oxoguanine, a byproduct of oxidative damage to guanine. Therefore, it was suggested that either the damage itself or the presence of OGG1 is responsible for the recruitment of the complex. Since guanine is easily oxidized and occurs in high concentrations in CpG islands, this offers a mechanism through which binding of the complex is increased in CpG islands compared to other regions. This differential binding of the silencing complex can potentially account for the observed changes in DNA methylation patterns in aging [4] and cancer [5], where non-CpG island are found to loose DNA methylation, while CpG islands experienced an increase in methylation. 

#### 4.4.4. ROS-Regulated Expression of *DNMT*s

Next to the previously described mechanisms, it appears that expression of the genes coding for DNMTs can be up-regulated by the superoxide anion (O_2_·-), a species of ROS [14]. In an *in vitro* experiment, Campos *et al.* [14] showed that during the transformation of a melanocyte cell line both oxidative stress and DNA methylation were enhanced. Upon further investigation, they discovered that as O_2_·- levels rose, so did *DNMT3B* and *DNMT1* expression. Inhibition of O_2_·- production resulted in the abolition of this expression, as well as the increase in DNA methylation. As these events coincided with the activation of RAS, the authors speculated that this regulation by O_2_·- might be mediated by the RAS signaling pathway, which is capable of activating the *DNMT* promoter [119]. 

In another *in vitro* study by Kang *et al.* [120], it was found that hydrogen peroxide (H_2_O_2_) decreased the expression of the tumor suppressor *RUNX1* gene in a colorectal cancer cell line by increasing the methylation of its promoter. Moreover, H_2_O_2_ induced the up-regulation of *DNMT1* and *histone deacetylase 1* (*HDAC1*), increased binding of DNMT1 to HDAC1 and increased binding of DNMT1 to the *RUNX1* promoter. It has been shown that DNMT1 and HDAC1 can associate to silence gene expression by deacetylation of the histones and methylation of the promoter [121,122]. This effect of H_2_O_2_ could be nullified by pretreatment with the ROS scavenger *N*-acytcysteine. 

The means by which these ROS can influence *DNMT* expression may involve a multistep process in which upregulation is attained through signaling pathways such as RAS. Currently, however, very little is known about the underlying mechanisms and more research is needed to identify the main players in this process. For example, it would be of interest to investigate to which extent this process occurs in healthy cells and which proteins involved could serve as potential drug targets.

#### 4.4.5. Other Mechanisms

The mechanisms described in the previous sections provide a substantial array of means by which ROS may influence DNA methylation. However, due to the high reactivity of these species, they are likely to influence many cellular processes. It is therefore possible that there are additional mechanisms by which ROS can elicit changes in the DNA methylome. For example, it has been shown that H_2_O_2_ is capable of inactivating Sirtuin-1 (SirT1), a histone deacetylase, by oxidizing critical cysteine residues in the enzyme [123]. Since SirtT1 has been shown to physically associate with DNMT1 and modulate its gene silencing activity [124], this offers yet another mechanism through which ROS can influence the methylome. Notably, it is known that an intricate interplay exists between histone modifications and DNA methylation (see [125]). Since ROS are capable of influencing both players in DNA methylation and histone modifications, it is not unlikely that they are responsible for shifts in both these epigenetic modifiers, which in turn may influence one another. Moreover, histone modification maps appear to also undergo distinct changes during transformation and with increasing age (see [126]). It might thus be of interest to study changes in histone modifications and DNA methylation upon exposure to ROS or during aging. 

## 5. Link to Disease

Evidently, there is a vast amount of evidence for the changing nature of the DNA methylome with increasing age and there are strong indications that ROS play a role in this process through various mechanisms. It is therefore of paramount importance to determine what the consequences of such modifications are for cellular processes and functioning. As DNA methylation is a strong determinant of gene expression, it is plausible that part of the effects of aging is exerted in this manner. Moreover, it has been shown already that changes in the methylome with age correspond to functional changes in gene expression [44]. It is therefore not surprising that new theories on aging have developed that highlight a more important role of epigenetic factors, such as DNA methylation [127], as reviewed by O’Sullivan and Karlseder [128]. 

Recently, research has been directed at how alterations of the methylome may contribute to the development of age-associated diseases. Although several of these diseases, such as Alzheimer’s disease, have been linked to changes in DNA methylation [129,130], cancer remains the most extensively studied and best understood example. The first indications of the involvement of altered DNA methylation in carcinogenesis already became apparent thirty years ago, when it was shown that cancerous cells could be distinguished from normal cells based on their DNA methylation levels [40]. Since then, proof has been accumulating for age-related changes in DNA methylation as a key player in carcinogenesis. Importantly, differential methylation patterns of cancerous cells seem to reflect those found in aging cells, with regional hypermethylation and general hypomethylation [5,7,131]. Such changes can contribute to the development of cancer in several ways. First of all, regional hypermethylation can result in the silencing of tumor suppressor genes [132,133]. Similarly, induced hypomethylation can result in the activation of proto-oncogenes [96,134], but also contributes to genomic instability and increased mutation rates [135,136]. For these reasons, age has been proposed to be an important contributor in the development of cancer and thus represents a major risk factor in carcinogenesis [8,9].

Another interesting research model to study the connection between differentially methylated CpG sites and aging is presented by cases of Hutchinson-Gilford Progeria and Werner syndrome. These diseases are defined by the occurrence of premature aging, resulting in aged phenotypes early in life [137]. The cause of these diseases appears to be genetic, as mutations in two particular genes are able to result in its onset [138,139]. However, there are also instances of these diseases in which no mutations are known. Recent research has shown that there is a connection between epigenetic disturbances and the observed symptoms [140,141], suggesting that these disturbances might be mediators of the apparent detrimental effects. In order to investigate the link between DNA methylation and both diseases, Heyn *et al.* [141] conducted a large scale study in which the methylation status of approximately 450,000 CpG loci in samples of patients was investigated. This showed that there were indeed CpG loci that were differentially methylated in diseased individuals as compared with healthy controls. Moreover, it was found that a subset of these loci corresponded to sites that become differentially methylated with age. These results suggest that there might be some CpG sites whose changing methylation status could be responsible for hallmarks of aging. Unsurprisingly, the affected CpG sites were enriched with promoters of genes that are likely to be involved in phenotypic changes observed during aging [141]. In addition to these findings, the researchers discovered that many of the promoters of differentially methylated genes were enriched with binding sites of transcription factor MYB in samples of patients without known mutations. Interestingly, this transcription factor has been linked to aging [142], as well as to the response to oxidative stress of aged cells [143]. 

These results indicate that changes to the DNA methylome might significantly contribute to age‑related changes in phenotype and risk for age-associated diseases. In particular, they exemplify the value of diseases such as cancer and premature aging disorders as research models in investigating the connection between DNA methylation and aging. 

## 6. Conclusion

Over the past decades, the molecular basis of aging has been extensively researched, resulting in a vast spectrum of theories. Thus far, the mitochondrial free radical theory of aging, proposed by Harman in 1956 [12], has proven the most fruitful and has promoted a lot of research regarding the connection between ROS and aging. Simultaneously, recent insights into regulation of DNA by methylation have resulted in the observation that the DNA methylome changes when organisms age. Most notably, specific regions of DNA hypermethylation appear to arise concurrently with a general genome-wide loss of methylation [4]. Since DNA methylation plays an important role in gene regulation, this could potentially explain the increase in risk for age-related pathologies and might even be involved in decreasing lifespan [8,9]. However, to date, a unifying theory that incorporates both the increase in ROS and changes in DNA methylation has been lacking. In this review paper, several mechanisms that could potentially link ROS to changes in DNA methylation have been discussed. For example, ROS‑induced DNA damage has been shown to modulate DNMT activity [99,100] and can alter the binding of DNMT-containing complexes [113]. Such events can eventually give rise to the observed shifts in DNA methylation. Research into these mechanisms might be able to clarify the link between current theories of aging and the changes in the methylome as well as explain changes in health and lifespan. 

In addition to identifying and understanding the ROS-dependent processes responsible for alterations in DNA methylation, it is also important to determine the frequency at which they happen *in vivo* and gain a better understanding to which extend they contribute to the age-related changes in DNA methylation. Particularly, other mechanisms have been proposed that may contribute to age‑related changes in DNA methylation. Such mechanisms include the altered transcriptional activity of DNA methyltransferases, in which DNMT1 appears to be down-regulated and DNMT3b up‑regulated [144,145]. Since DNMT1 is the predominant maintenance methyltransferase, this could account for the general levels of hypomethylation. Simultaneously, the *de novo* methyltransferase DNMT3b could thus be responsible for the site specific incidences in hypermethylation. Yet, the cause of these changes in transcriptional control remains unknown. Evidently, measuring the relative contributions of ROS-dependent and ROS-independent mechanisms is important when investigating both causes of aging and oncogenesis.

Animal models displaying different rates of aging and different levels of oxidative stress could considerably contribute to this line of research. The results of such efforts could lead the way towards means of interfering with these processes, both in aging and in cancer. Such a feat is difficult, however, since normally the accumulation of changes in the DNA methylome occurs over extended periods of time. Moreover, changes in DNA methylation and subsequently in gene expression could lead to clonal expansion of a subpopulation within a tissue, thereby distorting quantitative observations. As mentioned earlier, another important limitation is the fact that current genome-wide methylation profiling methods cannot differentiate between 5mC and 5hmC [107]. Considering the differential functions of hydroxymethylation as compared to methylation, this puts a substantial blind spot in the research of methylome dynamics over age. New screening methods that overcome this limitation thus have a big potential to spur research and increase understanding of both quantitative and qualitative changes in the DNA methylome.

Such insights into the aging methylome offer exiting opportunities, including early detection of age-related diseases and potentially even preventive therapy [146]. Therefore, new screening methods in combination with the recent advances in the field of DNA methylation and demethylation could open up new avenues for research and therapy and might eventually unveil the cause of aging. 
